# Autophagy inhibition of *hsa-miR-19a-3p/19b-3p* by targeting TGF-β R II during TGF-β1-induced fibrogenesis in human cardiac fibroblasts

**DOI:** 10.1038/srep24747

**Published:** 2016-04-21

**Authors:** Meijuan Zou, Fang Wang, Rui Gao, Jingjing Wu, Yingwei Ou, Xuguan Chen, Tongshan Wang, Xin Zhou, Wei Zhu, Ping Li, Lian-Wen Qi, Ting Jiang, Weiwei Wang, Chunyu Li, Jun Chen, Qifang He, Yan Chen

**Affiliations:** 1 Department of Pharmacology, School of Basic Medical Sciences, Nanjing Medical University, 140 Hanzhong Road, Nanjing 210029, P.R. China; 2 Department of Cardiology, First Affiliated Hospital of Nanjing Medical University, 300 Guangzhou Road, Nanjing 210029, P.R. China; 3 Department Of Nephrology, First Affiliated Hospital of Nanjing Medical University, 300 Guangzhou Road, Nanjing 210029, P.R. China; 4 Department of Oncology, First Affiliated Hospital of Nanjing Medical University, 300 Guangzhou Road, Nanjing 210029, P.R. China; 5 State Key Laboratory of Natural Medicines, China Pharmaceutical University, Nanjing 210009, China; 6 Emergency Center, First Affiliated Hospital of Nanjing Medical University, 300 Guangzhou Road, Nanjing 210029, P.R. China

## Abstract

Transforming growth factor-β1 (TGF-β1) plays an important role on fibrogenesis in heart disease. MicroRNAs have exhibited as crucial regulators of cardiac homeostasis and remodeling in various heart diseases. *MiR-19a-3p/19b-3p* expresses with low levels in the plasma of heart failure patients. The purpose of our study is to determine the role of *MiR-19a-3p/19b-3p* in regulating autophagy-mediated fibrosis of human cardiac fibroblasts. We elucidate our hypothesis in clinical samples and human cardiac fibroblasts (HCF) to provide valuable basic information. TGF-β1 promotes collagen I α2 and fibronectin synthesis in HCF and that is paralleled by autophagic activation in these cells. Pharmacological inhibition of autophagy by 3-methyladenine decreases the fibrotic response, while autophagy induction of rapamycin increases the response. BECN1 knockdown and Atg5 over-expression either inhibits or enhances the fibrotic effect of TGF-β1 in experimental HCF. Furthermore, *miR-19a-3p/19b-3p* mimics inhibit epithelial mesenchymal transition (EMT) and extracellular matrix (ECM) prodution and invasion of HCF. Functional studies suggest that *miR-19a-3p/19b-3p* inhibits autophagy of HCF through targeting TGF-β R II mRNA. Moreover, enhancement of autophagy rescues inhibition effect of *miR-19a-3p/19b-3p* on Smad 2 and Akt phosphorylation through TGF-β R II signaling. Our study uncovers a novel mechanism that *miR-19a-3p/19b-3p* inhibits autophagy-mediated fibrogenesis by targeting TGF-β R II.

Abnormal expression of cardiomyocyte gene can result in cardiomyocyte hypertrophy and impaired cardiomyocyte viability and contraction, ultimately resulting in heart failure (HF)^1^,^2^. The heart function decreases and affects the lungs, liver, and other body systems. HF is considered as the most common ultimate of many cardiovascular disease including dilated cardiomyopathy (DCM)^3^, myocardial infarction (MI)^4^,^5^, diabetic cardiomyopathy^6^,^7^, aortic stenosis (AS) and hypertension^8^,^9^. Interstitial fibrosis of myocardial cells may initiate with the dysfunctional cardiac remodeling following cardiac injury. Fibrosis is a complex process resulting from activation of some signaling pathways, such as Transforming growth factor (TGF)-β1 signaling^10^. Indeed, the dynamic mobilization within cardiac extracellular matrix (ECM) is critical during the pathogenesis of ventricular remodeling following DCM, MI, hypertension, and other cardiovascular conditions^11^. TGF-β1 signaling has broad-ranging effects that may affect cell growth, differentiation and the production of ECM proteins^12^,^13^,^14^.

TGF-β1 is also a known factor in angiotensin II (Ang II)-mediated cardiac fibrosis^15^. The phenomenon that an abundant latent collagenase system is closely associated with interstitial collagen matrix in heart has been identified for the first time by Montfort and Pérez-Tamayo in 1975^16^. During the harmful remodeling process, cardiac fibroblasts are differentiated into myofibroblasts and the ECM components such as collagen I α2 and fibronectin are accumulated^17^,^18^. Moreover, differentiation of fibroblasts into myofibroblasts activates matrix metalloproteinase (MMPs) such as MMP-2 and MMP-9 in the border of remodeling area. The MMPs activation accelerates degradation of adjoining ECM and thus facilitates the highly organized matrix to be replaced with the structureless and thickened matrix^19^,^20^. The dysregulation between accumulation and degradation of ECM has been involved in the mobility of ventricular geometry and function and then contributes to the development to heart failure (HF)^21^.

TGF-β Receptor II is formed with trans-membrane serine/threonine kinase and the TGF-β type II serine/threonine kinase receptor^22^. TGF-β Receptor II can transduce the TGF-β1, TGF-β2 and TGF-β3 signaling from cell membrane to cytoplasm and then regulate a series of physiological or pathological processes including mesenchymal cell proliferation and differentiation^23^,^24^, and ECM production^25^. Researchers have shown an association between a common TGF-β Receptor II polymorphism and risk of sudden cardiac arrest caused by ventricular arrhythmias in the setting of coronary artery disease^26^. The formation of the receptor complex composed of TGF-β Receptor I and TGF-β Receptor II molecules symmetrically bound to the cytokine dimer results in the phosphorylation and the activation of TGF-β Receptor I by the constitutively active TGF-β Receptor I^27^.

Autophagy works as a tightly-regulated process for bulk degradation, through which intracellular components are sequestered into autophagosomes and subsequently degraded by lysosomes^28^,^29^,^30^. Autophagy is critical for the clearance of damaged organelles and protein to maintain cellular homeostasis^31^,^32^. Autophagy can communicate with apoptosis as one of the programmed cell death through autodigestive cellular progression, cellular infection with pathogens or extracellular stimulation^29^,^30^,^33^,^34^. The overall regulation of interstitial fibrosis may contain the complex functioning of various regulatory factors^35^. It has been reported that the cardiomyocyte-specific deletion of basal autophagy resulting from Atg5 deficiency leads to spontaneous cardiac hypertrophy^36^,^37^. TGF-β1 can induce autophagy in other cellular systems, however, there is little evidence supporting the link between autophagy and fibrogenesis in cardiovascular disease.

MicroRNAs (miRNAs) are a series of small non-coding RNAs that regulate gene expression by binding to complementary sequences in un-translated regions (UTR) of target messenger RNAs (mRNAs) bearing fully complementary target sites to trigger either translation repression or mRNA degradation^38^,^39^. MiRNAs are involved in a wide spectrum of biological processes including autophagy^40^, fibrosis^41^, cell proliferation^42^, and apoptosis^43^. An abundant number of evidences indicate that miRNA-mediated gene negative regulation plays important roles in the cardiac homeostasis and pathological remodeling^44^,^45^,^46^,^47^. William T. Pu’s study has focused on the genome-wide miRNA expression profiling in left ventricular myocardium of 67 patients belonging to four diagnostic groups. The groups comprise ischemic cardiomyopathy (ICM), dilated cardiomyopathy (DCM), aortic stenosis (AS), and nonfailing controls^48^. They have found that miRNA expression profiles were significantly changed in heart diseases and that the pattern of miRNA expression was distinct in various forms of heart diseases, respectively. Among them, the expresson of *miR-19a-3p/19b-3p* is low in DCM (p ≦ 0.001), ICM (p < 0.001), and AS (p < 0.001)^48^. In our present study, we have found that expression of *miR-19a-3p/19b-3p* in HF patients is lower than in normal. In addition, the over-expression of *miR-19a-3p/19b-3p in vitro* sufficiently decreases the autophagy and fibrosis induced by TGF-β1 signaling, accompanied by inhibition effect of TGF-β R II. Our further investigation revealed the molecular mechanisms of *miR-19a-3p/19b-3p* in the regulation of autophagy-related fibrogenesis in human cardiac fibroblasts (which are responsible for the deposition of extracellular matrix).

## Method and Materials

### Cell culture and treatment

Human Cardiac Fibroblasts (HCF) was purchased from Cell Bank of Tongpai Biotechnology Co., Ltd. (Shanghai, China). The base medium for HCF cell line is formulated Dulbecco’s Modified Eagle’s Medium (DMEM, Gibco Inc.). To make the complete growth medium, we add the following components to the base medium: 4.5 g/L glucose and fetal bovine serum to a final concentration of 10%. Exponentially growing cultures were maintained in a humidified atmosphere of 5% carbon dioxide (CO_2_) at 37 °C.

### Cell proliferation assay

Cell growth was analyzed using the Cell Counting Kit-8 (Dojindo Molecular Technologies, Inc., Japan) according to the manufacturer’s instructions. Briefly, human Cardiac Fibroblasts were seeded at a density of 5 × 10^3 ^cells/well in 96-well plates and treated with TGF-β1 (10 ng/ml) for 0, 24, 48, 72, and 96 hours at 37 °C with 5% CO_2_. Then, the medium in each well was substituted with 100 μl of fresh medium containing 10% Cell Counting Kit-8, and the cultures were incubated at 37 °C for 2 hours. The absorbance value (A) was determined using Synergy™ 2 Multi-Mode Microplate Reader (BioTek Instruments, Inc., headquartered in Winooski, VT, USA) at 450 nm. Cell Viability (%) = average absorbance of treated group/average absorbance of control group ×100.

### *MiR-19a-3p/19b-3p* prediction targets

TargetScan 7.0 human, miRDB, miRanda, Ingenuity Pathway Analysis were used to identify predicted *miR-19a-3p/19b-3p* targets. The mRNAs would be considered as targets if selected miRNA was predicted with high probability to interact with their 3′-UTR. The mRNA targets were then compared to genes and pathways associated with human cellular morphology to evaluate potential miRNA regulation of cell motility.

### Dual-luciferase activity assay

The 3′UTR of human TGF-β R II cDNA containing the putative target site for the *miR-19a-3p/19b-3p* (sequence shown in Supplementary data) was chemically synthesized and inserted at the XbaI site, immediately downstream of the luciferase gene in the pGL3-control vector (Promega, Madison, WI) by Integrated Biotech Solutions Co., Ltd (Shanghai, China) (Supplementary data). Twenty-four hours before transfection, cells were plated at 1.5 × 10^5^ cells/well in 24-well plates. 200 ng of pGL3- TGF-β R II-3′-UTR plus 80 ng pRL-TK (Promega) were transfected in combination with 50 nM of the *miR-19a-3p/19b-3p* mimics or miRNA mimic control using Lipofectamine^TM^ 2000 reagent (Life Technologies, Carlsbad, CA, USA) according to the manufacturer’s protocol, respectively. Luciferase activity was measured 24 hours after transfection using the Dual Luciferase Reporter Assay System (Promega). Firefly luciferase activity was normalized to renilla luciferase activity for each transfected well. Three independent experiments were performed in triplicate.

The sequence of 3′UTR of human TGFBR2 cDNA containing the putative target site for the miR-19a-3p/19b-3p; stark black body stands for the putative target site for miR-19a-3p/19b-3p: CTCTTCTGGGGCAGGCTGGGCCATGTCCAAAGAGGCTGCCCCTCTCACCAAAGAACAGAGGCAGCAGGAAGCTGCCCCTGAACTGATGCTTCCTGGAAAACCAAGGGGGTCACTCCCCTCCCTGTAAGCTGTGGGGATAAGCAGAAACAACAGCAGCAGGGAGTGGGTGACATAGAGCATTCTATGCCTTTGACATTGTCATAGGATAAGCTGTGTTAGCACTTCCTCAGGAAATGAGATTGATTTTTACAATAGCCAATAACA**TTTGCAC**TTTATTAATGCCTGTATATAAATATGAATAGCTATGTTTTATATATATATATATATATCTATATATGTCTATAGCTCTATATATATAGCCATACCTTGAAAAGAGACAAGGAAAAACATCAAATATTCC

### The use of *miR19a/19b* mimics

The miR-Ribo^TM^ ***miR19a/19b*** mimics were chemically synthesized mature double stranded miRNA that could be ready to use (Guangzhou RiboBio Co., Ltd.). The product with lyophilized form was transported at normal temperature. The freeze-dried powder was prepared as a 20 μM stock solution in sterilized ddH_2_O and stored at −40 °C. For miRNA *miR19a/19b* mimics transfection, the optimal concentration for human cardiac fibroblasts was 50 nM. For 24-well plate system: 1.25 μl storage solution of miRNA mimic (20 μM) was diluted in 50 μl serum-free medium, mixed gently and incubated at room temperature for 5 minutes. For 6-well plate system: 5 μl storage solution of miRNA mimic (20 μM) was diluted in 250 μl serum-free medium, mixed gently and incubated at room temperature for 5 minutes.

### Transient transfections

Cells were transiently transfected with *miR-19a-3p/19b-3p* mimics, BECN1 siRNA, pCMV-myc-Atg5 plasmid using Lipofectamine^TM^ 2000 reagent (Life Technologies, Carlsbad, CA, USA), according to the manufacturer’s instructions^49^. Short interfering RNA against the human beclin 1 (BECN 1) and the sequence was: sense 5′-AAGAUUGAAGACACAGGAGGC-3′ and antisense 5′-GCCUCCUGUGUCUUCAAUCUU-3′. The universal negative control siRNA were used. The expression vector was transfected 24 hours before treatment with TGF-β1^30^.

### IF and confocal fluorescence microscopy

Human cardiac fibroblasts were pro-transfected with *miR-19a-3p/19b-3p* and then treated with 10 ng/ml TGF-β1. MAP-LC3 (green) was labeled with primary anti-MAP-LC3 polyclonal antibody. Goat anti-rabbit IgG/FITC were used as secondary antibody. Cells were simultaneously imaged in the presence of SNLYSO sensor (an autolysosome fluorescent probe, shown red) to visualize autophagy. The nuclei were stained with 4′, 6-diamidino-2-phenylindole (DAPI, Sigma-Aldrich, St. Louis, MO) for 10 minutes before imaging. An FV10-ASW laser scanning confocal microscope [Ver 2.1] (Olympus Corp, MPE FV1000) was used for co-localization analysis^30^.

### Total RNA extraction, Q-PCR for mRNA and miRNA quantification

Total RNA was extracted using Trizol reagent (Invitrogen, USA). The concentration and purity of the RNA samples were determined spectroscopically. Reverse transcription was performed with M-MLV (Promega, USA) following standard protocols. For the TaqMan-based real-time reverse transcription-polymerase chain reaction (RT-PCR) assays, the ABI 7900 HT Sequence Detection system (Applied Biosystem, Foster City, CA) was used. For quantitative PCR of miRNA, the *miR-19a-3p/19b-3p* primer and EzOmics SYBR qPCR kit were purchased from Biomics. Amplification procedure was 94 °C for 5 min, followed by 30 cycles at 94 °C for 30 s, 61 °C for 45 s, finally 72 °C for 10 min.

Variations in expression of *miR-19a-3p/19b-3p* between different RNA samples from cells or plasma were calculated after normalization to U6 or ath-miR-156a, respectively^50^. The methods were carried out in accordance with the approved guidelines and all experimental protocols were approved by the First Affiliated Hospital of Nanjing Medical University. The ethical code of our study is 2013-SRFA-078 obtained from the licensing committee of Nanjing Medical University. For quantification of TGF-β R II transcripts, Q-PCR was carried out with total RNA samples extracted from human cardiac fibroblasts after *miR-19a-3p/19b-3p* mimics transfection. TGF-β R II (158 bp) mRNA was amplified at an annealing temperature of 60 °C and using the following primers: TGF-β R II (forword): 5′-CAGAAATCCTGCATGAGC-3′ and TGF-β R II (reverse): 5′-GCAGCATCTTCCAGAATAAAG-3′. TaqMan quantitative assay was performed with the expression level of the corresponding housekeeping gene GAPDH as an internal control. The nucleotide sequences of *miR-19a-3p/19b-3p* are available in the GenBank database with the accession numbers of LM378760.1, LM378761.1.

### Cell synchronization and flow cytometry

Autophagy induction of human cardiac fibroblasts was examined using SNLYSO Autophagy Detection Kit (Catalog Number: E0010, SNPT, Chengdu, China), according to the manufacturer’s instructions. In brief, cells were treated for indicated condition and the medium was gently removed. 250 μl SNLYO Sensor was added to cover the sample and incubated for 20 hours. Finally, cells were treated with trypsin and collected to make the density of 10^5^~10^6^/ml and analyzed by BD FACSCalibur™ Flow Cytometry System (Franklin Lakes) and a computer station running Cell Quest software (BD Biosciences, Franklin Lakes, NJ).

### Transwell invasion assay

The invasion behavior of human cardiac fibroblasts was determined using 24-well Millicell Hanging Cell Culture inserts with 8 mm PET membranes (Millipore, Bedford, Massachusetts, USA) as described previously^45^,^51^,^52^,^53^. Briefly, after the cells were treated for indicated condition, 5.0 × 10^4^ human cardiac fibroblasts in 200 μl serum-free DMEM medium were plated onto BD BioCoat^TM^ Matrigel^TM^ Invasion Chambers (8 μM pore size polycarbonate filters; BD Biosciences), while complete medium containing 10% FBS was added to the lower chamber. After processing the invasion chambers for 48 hours (37 °C, 5% CO_2_) in accordance with the manufacturer’s protocol, the non-invading cells were removed with a cotton swab; the invading cells were fixed in 100% methanol and then stained with crystal violet solution and counted microscopically. The data are presented as the average number of cells attached to the bottom surface from five randomly chosen fields.

### Western blotting

Briefly, after washing twice with PBS, total cellular proteins were extracted with lysis buffer (50 mmol/l Tris [pH 7.4], 150 mmol/l NaCl, 1% Triton X-100, 1% deoxycholic phenylmethylsulfonyl fluoride, 1 mg/ml aprotinin, 5.0 mm sodium pyrophosphate, 1.0 g/ml leupeptin, 0.1 mm phenylmethylsulfonyl fluoride, and 1 mm/l DTT). Protease inhibitors were added immediately before use. The lysates were centrifuged at 13,000× g for 15 min at 4 °C. The concentration of total proteins was measured using the BCA assay method with Varioskan spectrofluorometer and spectrophotometer (Thermo, Waltham, MA) at 562 nm. Protein samples were separated with 12% SDS-PAGE gel and electrophoretically transferred onto the polyvinylidene difluoride (PVDF) membranes (Millipore, Boston, MA). Immune complexes were formed by incubation of proteins with primary antibodies overnight at 4 °C. After incubation with the appropriate secondary antibodies, blots were visualized using the ECL plus Western blotting detection reagents (Bio-Rad) and the ChemiDoc XRS Plus luminescent image analyzer (Bio-Rad, Hercules, CA, USA). Densitometric analysis of band intensity was performed using Image lab software (Bio-Rad, Hercules, CA, USA)^30^.

### Immunocytochemistry

IHC staining of heart valve tissue using MAP-LC3, TGF-β R I, TGF-β R II, Fibronectin, MMP-2, MMP-9 antibodies was performed. Briefly, the twenty heart valve tissue samples of 20 cases patients with dilated cardiomyopathy were subjected to deparaffinization, rehydration, and antigen retrieval before the staining procedures were performed. The tissue slides were blocked with 2.5% normal horse serum for 10 minutes. Then, tissue slides were incubated with rabbit anti-human MAP-LC3, TGF-β R I, TGF-β R II, Fibronectin, MMP-2, MMP-9 antibody (dilution 1:50) overnight at 4 °C. After the tissue slides were washed, they were incubated with anti-mouse IgG HRP and anti-rabbit IgG HRP secondary antibody for 10 minutes. The slides were stained with 3, 3′-diaminobenzidine (DAB) (Vector Laboratories), counterstained with hematoxylin (Vector Laboratories), dehydrated, treated with xylene, and mounted. All slides were examined and representative pictures were taken using an Olympus BX41 microscope (Olympus America, Melville, NY).

### Statistical analyses

All data were expressed as mean ± SEM and statistically compared by one-way ANOVA with Dunnett’s test and post-hoc tests were undertaken using the GraphPad Prism software. The details of each statistical analysis used were presented in the figure legends. Significance was indicated as *P < 0.05, ^#^P < 0.05 and **P < 0.01, ^##^P < 0.01, ^$$^P < 0.01.

## Results

### TGF-β1 simultaneously induces fibrosis and autophagy in human cardiac fibroblasts

**Figure 1:**
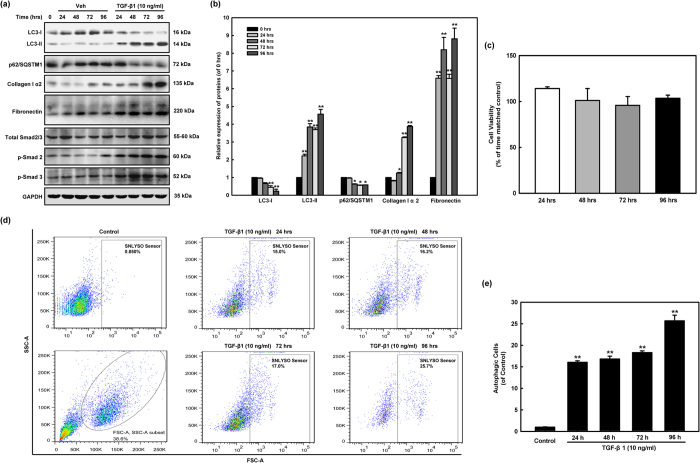
TGF-β1 simultaneously induces fibrosis and autophagy in human cardiac fibroblasts. **(a)** Human cardiac fibroblasts were treated with TGF-β1 (10 ng/ml) for 0, 24, 48, 72, and 96 hours. Protein samples were harvested for western blotting assay. The autophagy hallmarks (LC3-I, LC3-II, p62/SQSTM1), as well as indicator proteins of the fibrogenesis response in fibroblasts (collagen I α2, fibronectin and the Smad signaling pathway) were analyzed by western blotting assay. TGF-β1 induced LC3-II lipidation, with parallel increases in collagen Iα2, fibronectin expression and Smad2 and Smad3 phosphorylation. Data were normalized to GAPDH levels. Results are the means of three independent experiments from four different donors. **(b)** Representative quantitative data of densitometric analyses. Data are the means of three independent experiments from three different donors. For each experiment, LC3-I, LC3-II, p62/SQSTM1, collagen I α2, and fibronectin levels in HCF treated with TGF-β1 were compared with those from time-matched controls and normalized to GAPDH levels. *p < 0.05, **p < 0.01 vs. 0 hrs group. **(c)** TGF-β1 treatment does not affect cell viability of human cardiac fibroblasts. HCF cells were exposed to TGF-β1 (10 ng/ml) for the indicated time points (24, 48, 72, 96 hours), and cell viability and proliferation was measured by CCK-8 assay as described in the Materials and Methods section in three different culture experiments (n = 3). TGF-β1 treatment was not associated with any significant changes in cell viability. **(d,e)** HCF cells were treated with 10 ng/ml TGF-β1 for 0, 24, 48, 72, and 96 h. Cells were then probed by SNLYSO sensor (an autolysosome fluorescent probe) and autophagic cells were analyzed and quantitated by flow cytometer. Results from three independent experiments are shown as means ± SEM. **p < 0.01 vs. 0 hrs group.

Firstly, we investigated whether there was a correlation between TGF-β1-induced autophagy and fibrosis in human cardiac fibroblasts. Human cardiac fibroblasts were treated with TGF-β1 (10 ng/ml) for 0, 24, 48, 72, and 96 hours. Protein samples were harvested for western blotting assay. The autophagy hallmarks LC3-I, LC3-II, p62/SQSTM1, as well as indicator proteins of the fibrogenesis response Collagen I α2, Fibronectin and the Smad phosphorylation were analyzed. TGF-β1 induced LC3-II lipidation, with parallel increases in collagen I α2, fibronectin expression and Smad2 and Smad3 phosphorylation. Our results showed that TGF-β1 (10 ng/ml) significantly increased the synthesis of Collagen type I α2 and Fibronectin. Simultaneously, the conversion of water soluble MAP-LC3 (LC3- I) to the autophagosome-associated lipidated form (LC3-II) and p62 degradation were determined with the increase of Smad 2 and Smad 3 phosphorylation (Fig. 1a,b). The effect of TGF-β1 on cell cytotoxicity was determined by CCK-8 assay. As shown in Fig. 1c, we also showed that TGF-β1 treatment at a concentration of 10 ng/ml exhibited little effect on the viability of human cardiac fibroblasts. So, this dose was taken in the following experiments. To obtain more quantitative assessment of the induction of autophagy, we used flow cytometer analysis to count SNLYSO sensor (autolysosome fluorescent probe) labeled cells and assess the presence of autophagy flux. Figure 1d,e clearly show autophagosome and autophagolysosmes flux in human cardiac fibroblasts stimulated with TGF-β1.

### TGF-β1-induced autophagy is required for fibrosis in human cardiac fibroblasts

**Figure 2:**
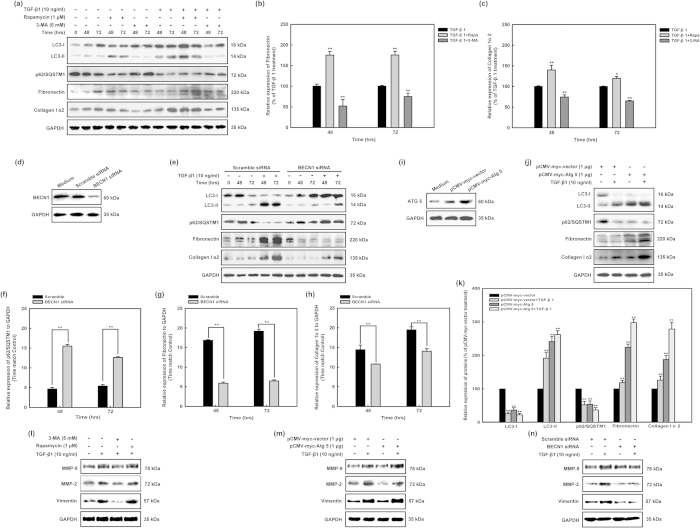
TGF-β1-induced autophagy is required for fibrogenesis in human cardiac fibroblasts. **(a)** HCF were treated with TGF-β1 (10 ng/ml) in the presence of the rapamycin (1μM) and 3-MA (5 mM) for indicated durations. Western blotting revealed that inhibition of autophagy by 3-MA abrogated fibrogenic effects of TGF-β1, whereas rapamycin treatment enhanced fibrogenic effects of TGF-β1. **(b,c)** Densitometric analysis of fibronectin and collagen I α2 levels in HCF, which were stimulated with TGF-β1 or rapamycin (1μM) and 3-MA (5 mM). **p < 0.01 vs. TGF-β1-treated group. **(d,e)** Inhibition of autophagy by BECN1 siRNA decreased fibronection and collagen I α2 biosynthesis. BECN1 knocked down cells and their correspondence scramble infected cells were treated with TGF-β1 (10 ng/ml) for 48 and 72 hours. Protein loading was confirmed using GAPDH. Data are means of independent experiments of HCF cells from three different donors. For each experiment, LC3-I, LC3-II, p62/SQSTM1, fibronectin, and collagen I α2 levels were compared with those from time-matched controls and normalized to GAPDH levels. **(f–h)** Densitometry analysis showed that BECN1 knockdown was associated with a significant (**p < 0.01) decrease of TGF-β1-induced fibronectin and collagen I α2 biosynthesis in HCF. **(I,j)** HCF were pro-transfected with Atg 5 (pCMV-myc-Atg 5) and TGF-β1 (10 ng/ml) for another 48 hours. LC3-I, LC3-II, p62/SQSTM1, collagen I α2, and fibronectin levels were measured in whole-cell lysates. **(k)** Densitometry analysis revealed that autophagy induction by Atg 5 overexpression was associated with a significant (**p < 0.01) increase in TGF-β1-induced fibrogenic effects in HCF. **(l)** HCF were treated with TGF-β1 (10 ng/ml) in the presence of the rapamycin (1μM) and 3-MA (5 mM) for 48 hours. MMP-9, MMP-2, and Vimentin levels were measured in whole-cell lysates and normalized to GAPDH levels. **(m,n)** HCF were pre-transfected with BECN1 siRNA or Atg 5 (pCMV-myc-Atg 5) and TGF-β1 (10 ng/ml) for another 48 hours. MMP-9, MMP-2, and Vimentin levels were measured in whole-cell lysates and normalized to GAPDH levels.

To further determine whether autophagy induction contribute to TGF-β1-induced fibrosis, we examined the effects of TGF-β1 stimulation of human cardiac fibroblasts in the presence of autophagy inducer and inhibitor. Rapamycin and 3-methyladenine (3-MA) are known pharmacological inducer and inhibitor of autophagy, as reported by our group and others. Thus we co-treated human cardiac fibroblasts with rapamycin (1 μM) or 3-MA (5 mM) and TGF-β1 (10 ng/ml) for 48 and 72 hours, and collagen type I α2 and fibronectin expression levels were then compared with their corresponding controls (Fig. 2a,b). We found that rapamycin or 3-MA co-treatment significantly increased or decreased TGF-β1-induced pro-fibrotic effects in human cardiac fibroblasts (Fig. 2b). Densitometric analysis of LC3-II in co-treated human cardiac fibroblasts (TGF-β1 and autophagy inducer or inhibitor) versus controls revealed that TGF-β1 treatment is associated with an increase in LC3 -II at 48 hours versus control and an increase in LC3-II at 96 hours. We suggest that increased or decreased autophagy in the presence of autophagy inducer or inhibitor is linked to basal production of matrix component proteins and thus that autophagy is positively correlated to the synthesis of matrix components and human cardiac fibroblasts function.

Moreover, to utilize a parallel non-pharmacological approach to test the same hypothesis, beclin 1 (BECN1) gene expression was suppressed using siRNA interference in human cardiac fibroblasts (Fig. 2c) and later treated with TGF-β1 (10 ng/ml). These cells were compared with human cardiac fibroblasts, which were infected with scrambled siRNA. The expression of collagen type I α2 and fibronectin were then detected and compared between groups of scrambled siRNA and BECN1 siRNA transfection. The results showed that BECN1 knockdown significantly decreased TGF-β1-induced pro-fibrotic effects (Fig. 2d–g). We further used pCMV-myc-Atg5 plasmid to transfect into human cardiac fibroblasts and showed that TGF-β1 stimulation induced significantly more fibronectin synthesis in pCMV-myc-Atg5 overexpressed cells compared with corresponding control cells (Fig. 2h–k). Our results showed that rapamycin and pCMV-myc-Atg5 increased TGF-β1-induced expression of MMP2, MMP9, and Vimentin in human cardiac fibroblasts (Fig. 2l,m); 3-MA and BECN1 siRNA decreased TGF-β1-induced expression of MMP2, MMP9, and Vimentin in human cardiac fibroblasts (Fig. 2l,n), respectively. Our experiments showed that autophagy induction significantly increased the TGF-β1-induced fibrogenic effect.

### Coincidence of elevated autophagy marker and fibronectin induction in human heart valve tissue

**Figure 3:**
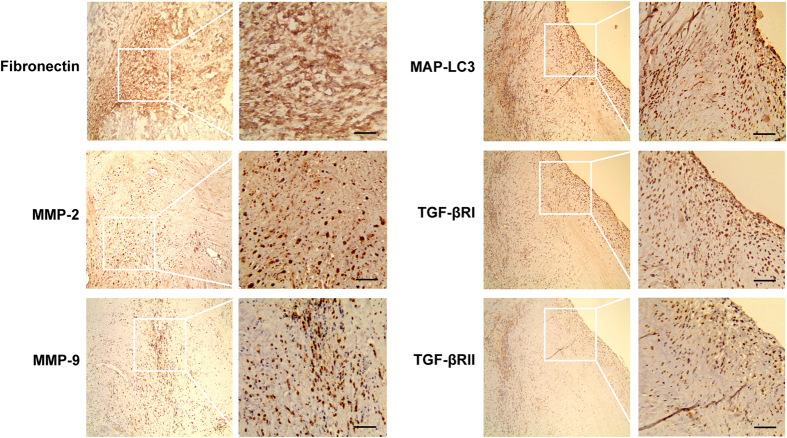
Coincidence of elevated autophagy, fibrosis, invasion and TGF-β RI/II in human heart valve tissue. The twenty heart valve tissue samples of 20 cases patients with dilated cardiomyopathy were randomly selected for IHC assay. Representative photomicrographs of Fibronectin, MMP-2, MMP-9, MAP-LC3, TGF-β R I, and TGF-β R II expression in 20 samples of heart valve tissue from patients with dilated cardiomyopathy were determined by IHC staining. Image magnification: 100× (left); 200× (right). Scale bar of 10 mm (right) is equivalent to 50 μm.

The twenty heart valve tissue samples of 20 cases patients with dilated cardiomyopathy were randomly selected for IHC assay. Representative photomicrographs of Fibronectin, MMP-2, MMP-9, MAP-LC3, TGF-β R I, and TGF-β R II expression in 20 samples of heart valve tissue from patients with HF were determined by IHC staining. Our results showed in Fig. 3 that heart valve tissue from patients with dilated cardiomyopathy was positive for Fibronectin, MMP-2, MMP-9, MAP-LC3, TGF-β R I, and TGF-β R II, except for sparse cytoplasmic staining in a few glia and neurons. Although these experiments were only conducted in specimens from a limited number of patients, their results are consistent with the preclinical evidence shown above and suggest that notably high expression of TGF-β R I and TGF-β R II may be associated with autophagy-mediated fibrosis in human heart diseases.

### *MiR-19a-3p/19b-3p* expresses with low levels in the plasma of HF patients

Studies of the last two years have shown that miRNA can be free outside the cells and stable in plasma or serum. Thus, these miRNAs can be used as disease diagnosis, therapeutic response and prognosis evaluation of effective and sensitive biomarker^54^. Thus, the differential expressions of 26 kinds of microRNAs from the literature were verified by Q-PCR assay and ath-156a was added as the external reference for corresponding control. Results show that expression of miRNA19a-3p/19b-3p is much low in HF patients (Fig. S1a,b). As shown in Fig. S1c,d, the expressions of miRNA 19a-3p/19b-3p were significantly down-regulated in DCM plasma pool samples. The expression of miRNA 19a-3p/19b-3p in the plasma samples of 38 patients with DCM was quantitatively tested by qRT-PCR. MiRNA 19a-3p/19b-3p decreased in DCM plasma samples. P < 0.001 (miRNA 19a); P = 0.001 (miRNA 19b). The expression of miRNA 19a-3p/19b-3p decreased more obviously in end stage of DCM plasma samples than in early stage. P = 0.009 (miRNA 19a); P = 0.008 (miRNA 19b) (Fig. S1e,f).

**Figure 4:**
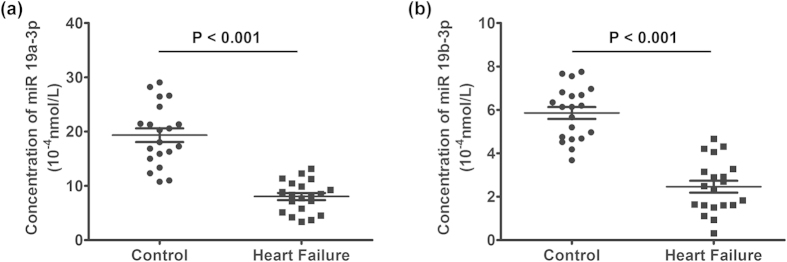
*MiR-19a-3p/19b-3p* expresses with low levels in heart failure patients. **(a,b)** The plasma expression levels of *miR-19a-3p/19b-3p* in heart failure (HF) and Control groups. The y-axis represents the miRNA’s concentration (1 × 10^−4 ^nM) calculated after normalization to ath-miR-156a, which served as external reference. P < 0.001.

In addition, twenty plasma samples of 20 cases patients with heart failure were randomly selected as HF plasma samples; and another twenty plasma samples of 20 cases normal people were selected as normal control plasma samples. The characteristics of heart failure patients (HF group) and healthy controls (C group) were displayed in (Table 1). As shown in Fig. 4a,b, the expression of *miR-19a-3p/19b-3p* was significantly down-regulated in HF plasma samples, P < 0.001 compared to Control group.

### *MiR-19a-3p/19b-3p* negatively regulates multiple players in fibrosis

**Figure 5:**
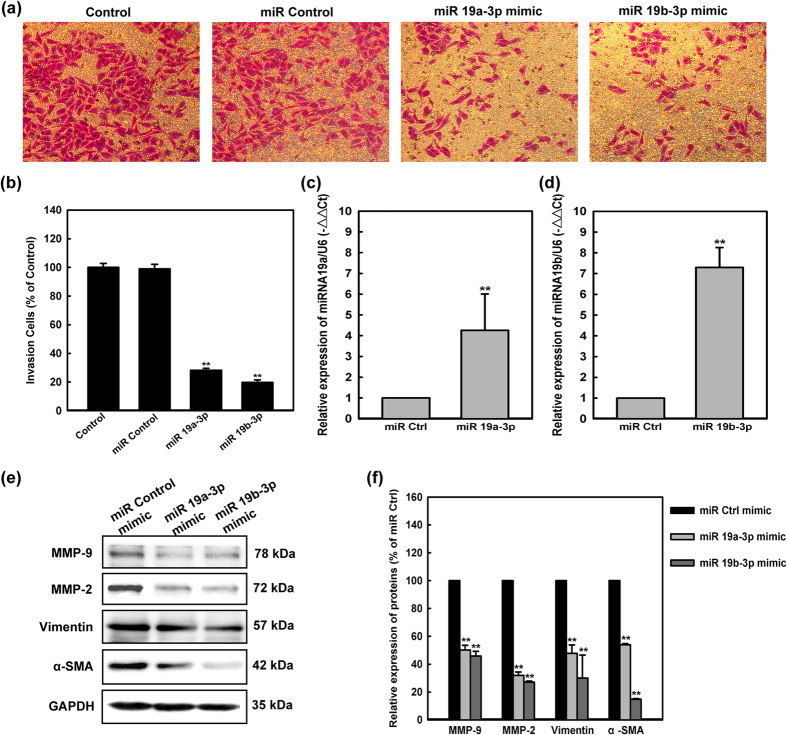
*MiR-19a-3p/19b-3p* negatively regulates multiple players in fibrosis. **(a)** Photographs of the cell invasion through the polycarbonate membrane stain by crystal violet. The migratory cell numbers of human cardiac fibroblasts transfected with *miR-19a-3p/19b-3p* mimics were significantly more than that of cells transfected with miR Control respectively. **(b)** The inhibitory effect of *miR-19a-3p/19b-3p* on the invasion of the cells was quantified. **P < 0.01 compared with control. Data were presented as the mean ± SEM of three separate experiments. **(c,d)** The Q-PCR analyses of the expression of *miR-19a-3p/19b-3p* in human cardiac fibroblasts transfected with *miR-19a-3p/19b-3p* mimics, NC and untranfected one. −△△Ct = −(Ctx-CtU6x)-(CtNCx-CtU6_NC_). Data were present as mean ± SEM, n = 3, **P < 0.01 vs. NC group. **(e)** Human cardiac fibroblasts were transfected with *miR-19a-3p/19b-3p* mimics, miR Control. Expression of MMP-2, MMP-9, Vimentin and α-SMA after 72 hours were detected by western blotting. Protein loading was confirmed using GAPDH. **(f)** Quantitative data of densitometric analyses. The ratio of MMP-2, MMP-9, Vimentin and α-SMA to GAPDH were displayed as mean ± SEM, n = 3, *p < 0.05, **p < 0.01 vs. NC group.

To investigate whether the differential expression of *miR-19a-3p/19b-3p* was correlated with cell invasion, human cardiac fibroblasts were transfected with *miR-19a-3p/19b-3p* mimics and nonspecific miRNA Control. Transwell migration assay was used to investigate migration activity. As shown from the images in Fig. 5a,b, when the expression of *miR-19a-3p/19b-3p* was up-regulated by mimics in human cardiac fibroblasts, the cells demonstrated much low-migration potentiality compared to cells treated with miRNA Control. Q-PCR was used to analyze the expression of *miR-19a-3p/19b-3p* when treated with mimics and nonspecific miRNA Control. The result showed that the expression of *miR-19a-3p/19b-3p* was negative correlation with the migration ability of human cardiac fibroblasts (Fig. 5c,d).

It has been reported that MMP-2 and MMP-9 play a critical role in cell invasion by stimulating degradation of the ECM and cell migration. To probe the possible anti-invasion mechanism of *miR-19a-3p/19b-3p*, we tested the activity protein of MMP-2/9 in *miR-19a-3p/19b-3p*-transfected human cardiac fibroblasts. The protein of MMP-2 and MMP-9 in were decreased by *miR-19a-3p/19b-3p*, suggesting that *miR-19a-3p/19b-3p* suppresses the invasion ability of human cardiac fibroblasts through down-regulation of the expression of MMP-2/9 (Fig. 5e,f). Epithelial mesenchymal transition (EMT), characterized as loss of polarity and epithelial markers (including junctional and cell-cell adhesion proteins), has long been known to play a role in cellular metastasis and cell invasion. To further examine whether *miR-19a-3p/19b-3p* would inhibit EMT, consistent with metastasis ability, after transient transfection with *miR-19a-3p/19b-3p* mimics in human cardiac fibroblasts, both vimentin and α-SMA mRNA levels were decreased compared to miRNA Control group (Fig. 5e,f). In summary, *miR-19a-3p/19b-3p* was important in regulating reorganization of actin cytoskeleton and the maintenance of cell morphology.

### *MiR-19a-3p/19b-3p* directly targeted TGF-β Receptor II

**Figure 6:**
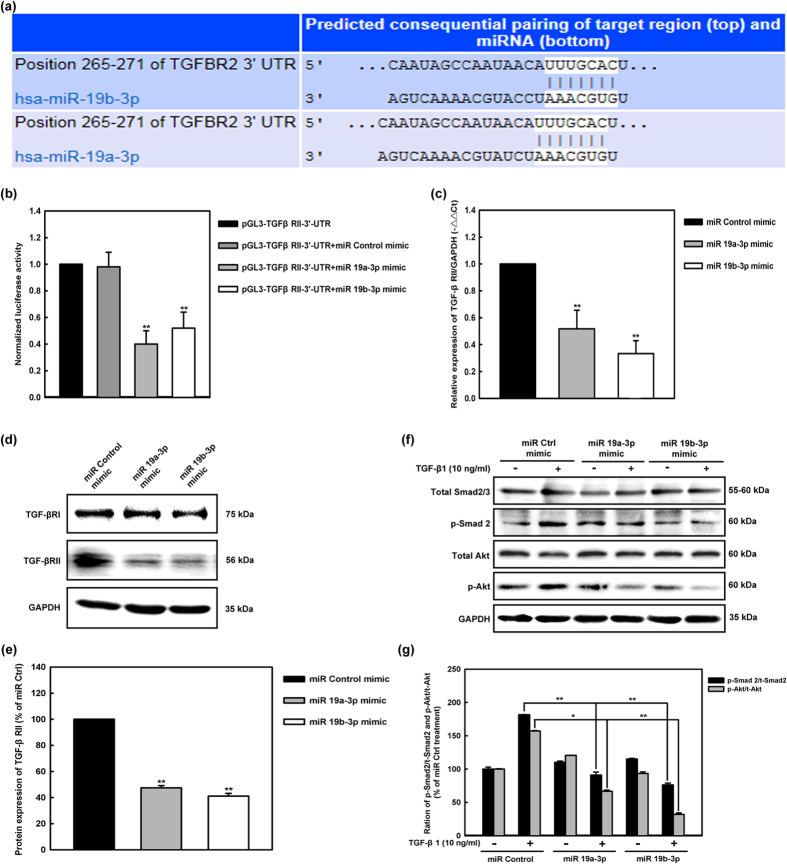
*MiR-19a-3p/19b-3p* directly targets TGF-β Receptor II. **(a)** The miR-19a/b directly target on human TGF-β Receptor II were predicted using TargetScan 7.0. **(b)** Dual luciferase assay performed in HCF cells suggested that TGF-β Receptor II was the target gene of the *miR-19a-3p/19b-3p*. In HCF cells, significant decrease in relative luciferase activity was noted when pGL3- TGF-β R II -3′-UTR was cotransfected with the *miR-19a-3p/19b-3p* mimics but not with the miRNA mimic control, respectively. **p < 0.01. **(c)** TGF-β Receptor II mRNA levels were determined by Q-PCR and normalized to GAPDH. Human cardiac fibroblasts were transfected with *miR-19a-3p/19b-3p* mimics, miR Control. The gene expression of TGF-β Receptor II was detected after 48 hours. Data were present as the mean ± SEM, n = 3, **p < 0.01 vs. miR Control group. (**d)** Human cardiac fibroblasts were transfected with *miR-19a-3p/19b-3p* mimics, miR Control. TGF-β Receptor I/II protein and GAPDH after 72 hours were detected by western blotting. **(e)** Quantitative data of densitometric analyses. *MiR-19a-3p/19b-3p* mimic treatment was not associated with any significant changes in TGF-β Receptor I expression. The ratio of TGF-β Receptor II protein to GAPDH were displayed as mean ± SEM, n = 3, **p < 0.01 vs. miR Control group. **(f)** Western blotting analyses for total and phosphorylated forms of Smad 2 and Akt (p-Smad 2, p-Akt) expression in human cardiac fibroblasts. The experiments were normalized with GAPDH at baseline level and after pro-transfection with *miR-19a-3p/19b-3p* mimics, miR Control and stimulation with TGF-β1 (10 ng/ml) for 72 hours. Results are the means from three independent experiments using cells from three different donors. **(g)** Densitometric analysis of Smad2 and Akt phosphorylation levels in human cardiac fibroblasts. Each bar represents the mean ± SEM calculated from three independent experiments. *p < 0.05 compared with miR Control and TGF-β1 group; **p < 0.01 compared with miR Control and TGF-β1 group.

It is generally considered that miRNAs exert their function through regulating the expression of their downstream target genes. To investigate the target of *miR-19a-3p/19b-3p* in human cardiac fibroblasts, systemic bioinformatic publicly available algorithms were used to analyze and identify potential targets. We found that human TGFBR2 (TGF-β Receptor II) 3′-UTR contained putative *miR-19a-3p/19b-3p* complementary sites predicted using TargetScan 7.0 (http://www.targetscan.org) (Fig. 6a). To explore whether the TGF-β Receptor II was the target gene of the *miR-19a-3p/19b-3p*, we constructed the luciferase reporter vector with the putative TGF-β Receptor II 3′ UTR target site for the *miR-19a-3p/19b-3p* downstream of the luciferase gene (pGL3- TGF-β R II -3′-UTR). Luciferase reporter vector together with the *miR-19a-3p/19b-3p* mimics or the miRNA mimic control were transfected into HCF, respectively. In HCF, significant decrease in relative luciferase activity was noted when pGL3- TGF-β R II -3′-UTR was cotransfected with the *miR-19a-3p/19b-3p* mimics but not with the miRNA mimic control, respectively. These results showed that TGF-β R II was the target gene of the *miR-19a-3p/19b-3p* (Fig. 6b). To further identify the *miR-19a-3p/19b-3p* putative target gene TGF-β R II that might be involved in autophagy-related fibrosis, transfection and Q-PCR analysis was conducted to determine the mechanism by which *miR-19a-3p/19b-3p* inhibited TGF-β Receptor II mRNA. Figure 6c indicates that transfection of human cardiac fibroblasts from controls with *miR-19a-3p/19b-3p* mimics induced significant decrease in TGF-β Receptor II mRNA expression levels compared to miRNA Control transfection. Western blotting revealed that the protein level of TGF-β Receptor II but not TGF-β Receptor I was markedly reduced in the cells over-expressing *miR-19a-3p/19b-3p* compared to the non transfected cells (Fig. 6d,e).

We further evaluated whether *miR-19a-3p/19b-3p* modulation altered the activation of Smad/Akt pathway proteins after TGF-β1 treatment. Western blotting analyses for total and phosphorylated forms of Smad 2 and Akt (p-Smad 2, p-Akt) expression in human cardiac fibroblasts were carried out. The experiments were normalized with GAPDH at baseline level and after pro-transfection with *miR-19a-3p/19b-3p* mimics, miR Control and stimulation with TGF-β1 (10 ng/ml) for 72 hours. As shown in Fig. 6f, the functional phosphorylation level of Smad 2 and Akt is up-regulated by TGF-β1 treatment in human cardiac fibroblasts. *MiR-19a-3p/19b-3p* modulation did not affect the expression of total Smad 2/3 and Akt. However, the over-expression of *miR-19a-3p/19b-3p* significantly reduced the level of the phosphorylated forms of Smad 2 and Akt (Fig. 6f,g).

### *MiR-19a-3p/19b-3p* decreases TGF-β1 induced autophagy-related fibrosis

**Figure 7:**
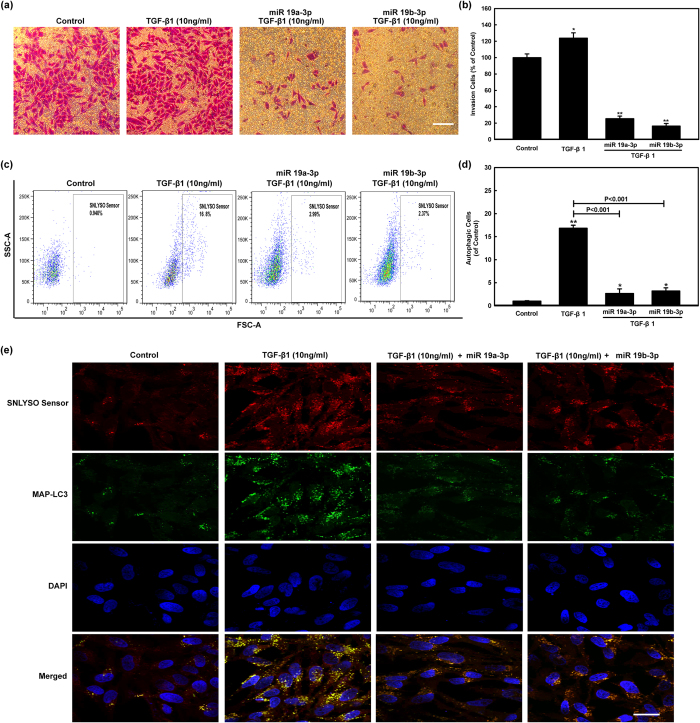
*MiR-19a-3p/19b-3p* regulates human cardiac fibroblasts autophagy-mediated fibrosis induced by TGF-β1. **(a)** Effects of *miR-19a-3p/19b-3p* and TGF-β1 on cell invasion in human cardiac fibroblasts *in vitro*. Photographs of the cell invasion through the polycarbonate membrane stained with crystal violet. **(b)** The inhibitory effect of *miR-19a-3p/19b-3p* and TGF-β1 on the invasion of the cells was quantified. Data were presented as the mean ± SEM of three separate experiments. *p < 0.05, **p < 0.01, group of pro-transfected with *miR-19a-3p/19b-3p* mimics and stimulation of TGF-β1 compared with control. **(c,d)** Human cardiac fibroblasts were pro-transfected with *miR-19a-3p/19b-3p* and then treated with 10 ng/ml TGF-β1 for 72 h. Cells were then probed by SNLYSO sensor and autolysosomes were analyzed and quantitated by flow cytometer. Results from three independent experiments are shown as means ± SEM. **p < 0.01, group of pro-transfected with *miR-19a-3p/19b-3p* mimics and stimulation of TGF-β1 compared with control. **(e)** Confocal fluorescence images of endogenous MAP-LC3 and SNLYSO sensor-labeled compartment in human cardiac fibroblasts. Human cardiac fibroblasts were pro-transfected with *miR-19a-3p/19b-3p* and then treated with 10 ng/ml TGF-β1. MAP-LC3 (green) was labeled with primary anti-MAP-LC3 polyclonal antibody. Goat anti-rabbit IgG/FITC were used as secondary antibody. Cells were simultaneously imaged in the presence of SNLYSO sensor (red) to visualize autophagy. Nuclei (blue) were labeled by DAPI. Confocal microscopy images were obtained. Bar = 30 μm.

We validated that *miR-19a-3p/19b-3p* negatively regulates multiple players in autophagy and fibrosis and that TGF-β R II was one of *miR-19a-3p/19b-3p* targets was also validated in previous results. To further explore whether *miR-19a-3p/19b-3p* inhibited autophagy-mediated fibrosis induced by targeting TGF-β1, pharmacological induction of autophagy was applied. It was shown in Fig. 7 that we got an expected inhibition effect after transfected with *miR-19a-3p/19b-3p* for 72 h. The invasion potential of human cardiac fibroblasts was examined by transwell invasion assay with co-treatment of *miR-19a-3p/19b-3p* mimics and TGF-β1 (10 ng/ml). The treatment with *miR-19a-3p/19b-3p* mimics led to significantly decreased invasion cells compared to the treatment of Control group (Fig. 7a,b). Further we investigated the relationship between autophagy and invasion potential in human cardiac fibroblasts by SNLYSO autophagy detection assay with co-treatment of *miR-19a-3p/19b-3p* mimics and TGF-β1 (10 ng/ml). It was shown in Fig. 7c,d, the autophagy induction of TGF-β1 was significantly reduced by *miR-19a-3p/19b-3p* mimics (16.8% to 2.37% and 2.99%). Confocal fluorescence images of endogenous MAP-LC3 and SNLYSO sensor-labeled compartment in human cardiac fibroblasts also verified the above results. The results showed that *miR-19a-3p/19b-3p* reduced the accumulation of both the yellow and red puncta induced by TGF-β1 (Fig. 7e). The vacuoles assumed to be autophagosomes, would be expected to undergo acidification after maturation and finally, fuse with lysosomes so that their content is digested by lysosomal hydrolases. The large endosomes subsequently recruit multiple autophagosomes.

**Figure 8:**
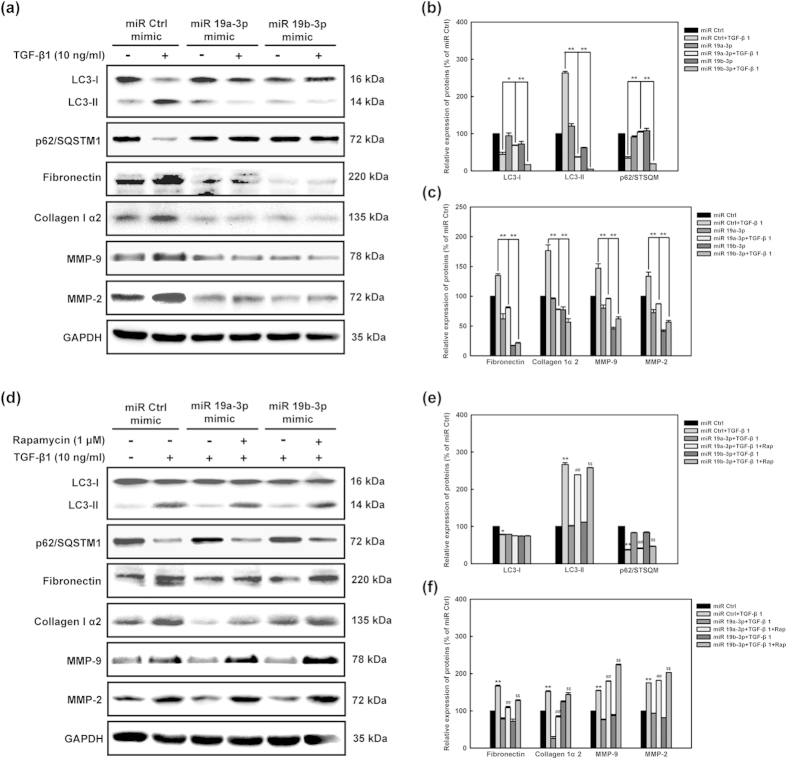
*MiR-19a-3p/19b-3p* decreases expression of autophagy-related fibrosis members induced by TGF-β1. **(a)** Human cardiac fibroblasts were pre-transfected with *miR-19a-3p/19b-3p* and miR Control. Then cells were treated with TGF-β1 (10 ng/ml) for the indicated duration. LC3-I, LC3-II, p62/SQSTM1, collagen I α2, fibronectin, MMP-9, and MMP-2 levels were measured in whole-cell lysates. Protein loading was confirmed using GAPDH. **(b,c)** Quantitative data of densitometric analyses. The ratios of LC3-I, LC3-II, p62/SQSTM1, collagen I α2, fibronectin, MMP-9, and MMP-2 to GAPDH were present as mean ± SEM, n = 3, *p < 0.05, **p < 0.01 vs. group of miR Control and TGF-β1 co-treatment. **(d)** Rapamycin reverses the inhibition effect of *miR-19a-3p/19b-3p* on TGF-β1-induced autophagy-related fibrosis. Human cardiac fibroblasts were transfected with *miR-19a-3p/19b-3p* and miR Control. Then cells were pretreated with rapamycin (4 hours, 1 μM) and co-treated with TGF-β1 (10 ng/ml) for the indicated duration. LC3-I, LC3-II, p62/SQSTM1, collagen Iα2, fibronectin, MMP-9, and MMP-2 levels were measured in whole-cell lysates. Protein loading was confirmed using GAPDH. **(e,f)** Densitometry analysis showed that rapamycin (1 μM) significantly reversed the inhibition of *miR-19a-3p/19b-3p* on TGF-β1-induced autophagy-related fibrosis in human cardiac fibroblasts. *p < 0.05, **p < 0.01 vs group of miR Control and TGF-β1 co-treatment; ^##^p < 0.01 vs group of miR 19a-3p and TGF-β1 co-treatment; ^$$^p < 0.001 vs group of miR 19b-3p and TGF-β1 co-treatment.

We next determined whether the expression of autophagy and fibrosis related proteins would be modulated by *miR-19a-3p/19b-3p*. Human cardiac fibroblasts were pre-transfected with *miR-19a-3p/19b-3p* and miR Control. Then cells were treated with TGF-β1 (10 ng/ml) for the indicated duration. LC3-I, LC3-II, p62/SQSTM1, collagen Iα2, fibronectin, MMP-9, and MMP-2 levels were measured in whole-cell lysates. Protein loading was confirmed using GAPDH. The conversion from LC3-Ito LC3-II and degradation of p62/SQSTM1 induced by TGF-β1 were significantly restored by *miR-19a-3p/19b-3p* (Fig. 8a,b). Consistently, the increased synthesis of collagen type Iα2 and fibronectin and expression of MMP-2 and MMP-9 induced by TGF-β1 were also significantly restored by *miR-19a-3p/19b-3p* (Fig. 8a,c). To determine whether autophagy activation rescued the inhibition effect of *miR-19a-3p/19b-3p* on autophagy-related fibrosis, rapamycin was applied for autophagy inducer. As is shown in Fig. 8d–f, rapamycin could further increase LC3-II level, synthesis of collagen type I α2 and fibronectin and expression of MMP-2 and MMP-9 in human cardiac fibroblasts with *miR-19a-3p/19b-3p* and TGF-β1. Taken together, these results demonstrated that autophagy mediated the regulation of *miR-19a-3p/19b-3p* on fibrosis in human cardiac fibroblasts.

## Discussion

HF is characterized by left ventricular (LV) remodeling and dilatation, with activation of a fetal gene program that triggers pathological changes in the myocardium associated with progressive dysfunction^55^. Tissue biopsy and cardiac radionuclide scan showed that there was no necrosis of cardiac muscle cells in the early stage of HF, but the degeneration, apoptosis and interstitial fibrosis of myocardial cells^56^,^57^,^58^,^59^,^60^,^61^. Therefore, most of the early HF patients firstly exhibit heart cavity expansion without obvious cardiac function insufficiency. With the continued existence of pathological matrix, necrosis of myocardial cells and extracellular matrix edema begin to appear, and then the heart function reduces till heart failure. Therefore, further study on the internal mechanism of HF pathology and explore the effective means to block the mechanism, will provide valuable basic research information for clinical treatment of HF. Indeed, certain mutations often induce HF with cardiac arrhythmia that is considered as the potential trigger of sudden cardiac death^62^,^63^. Thus, both the effective prognostic determination and appropriate cardiac care depend on accurate molecular and genetic diagnoses and therapy.

In this study, we have shown that TGF-β1 treatment simultaneously induced autophagy and fibrosis in human cardiac fibroblasts (Fig. 1). Either inhibition or induction of autophagy reduces and increases fibrotic characteristic in TGF-β1-induced fibrosis (Fig. 2). These findings highlighted a linking between autophagy and elevated matrix protein synthesis by human cardiac fibroblasts and strongly supported the hypothesis that TGF-β1-induced fibrosis was depending on its autophagy induction. In the present study we showed that the over-expressions of TGF-β Receptor I and TGF-β Receptor II are accompanied with hallmarks of autophagy, fibrosis, and ECM production in human cardiac tissues from heart valve (Fig. 3). Although miRNAs are essential to the regulation of cardiac remodeling, many of the mechanisms have not been well characterized^45^,^46^,^47^,^64^. The *miR-19a-3p/19b-3p* expression is specifically decreased in patients with DCM, especially in the end stage of DCM (Fig. 4). Herein we demonstrated that the cardiac-specific over-expression of *miR-19a-3p/19b-3p* resulted in inhibition of interstitial fibrosis, invasion potential, and epithelial mesenchymal transition (EMT) (Fig. 5). Furthermore, we confirmed and extended our previous discoveries, revealing that *miR-19a-3p/19b-3p* is a negative regulator of TGF-β1/Smad2 signaling via directly targeting TGF-β Receptor II (Fig. 6). To elucidate that *miR-19a-3p/19b-3p* is the important regulator of cardiac homeostasis and remodeling for potential therapeutic strategy for heart failure, we therefore demonstrated the *miR-19a-3p/19b-3p* inhibited the fibrosis through autophagy inhibition by targeting TGF-β R II in human cardiac fibroblasts (Figs 7 and 8).

Autophagy basically exists in most cells and can be rapidly activated as an adaptive response to renew intracellular substances and nutrition under cellular stimulation^65^,^66^. Autophagy (macro-autophagy) is a highly conserved process that is tightly regulated by various biological mechanisms and has important roles in many events, such as cellular remodeling during differentiation, development, adapt to environmental stress^31^,^67^. In contrast, autophagic cell death can drive cells towards type II programmed cell death, which is morphologically distinct from type I programmed cell death (apoptosis). However, a growing body of evidences points that autophagy is more important for cell survival. The accumulation of autophagosomes and autolysosomes exhibit survival response to deadly stress in order to rid the cell of harmful proteins or damaged organelles^33^,^65^. Autophagy is required to maintain cardiac homeostasis and function. Conversely, disrupted autophagy may contribute to cardiac remodeling^37^. TGF-β1 has been demonstrated to induce both autophagy and fibrosis in many tissues^35^,^68^. The coincidence of elevated autophagy marker and fibronectin induction in many diseases has been previously observed^35^. In the present study, our results demonstrate that autophagy is necessary for induction of fibrosis in human cardiac fibroblasts.

After TGF-β1 binding to the TGF-β R II, the phosphorylated TGF-β R I recruits and phosphorylates receptor-regulated Smad proteins (Smad2/3 complex). Thus, we have evaluated the functional phosphorylated form of Smad 2 and Smad 3 after TGF-β1 treatment and *miR-19a-3p/19b-3p* modulation. Our data show that the phosphorylated activated form of Smad 2 and Smad 3 after *miR-19a-3p/19b-3p* over-expression. Therefore the down-regulation of TGF-β R II and phosphorylated form of Smad 2 and Smad 3 are modulated by *miR-19a-3p/19b-3p*.

MiRNAs are considered as important regulators of gene expression, suppressing the expression of target genes through translational repression or degradation of a target transcript^39^,^53^. Because of the difficulty to obtain clinical HF heart tissue, we could not verify the miRNA expression in HF heart tissues. However, an important study on heart tissues of 25 patients with HF has shown the abnormal expression of various miRNA. Among them, the decreasing ratio of miRNA-19a/b is the most distinct^48^. Here, we focused on the regulation of *miR-19a-3p/19b-3p* on the autophagy-related fibrosis in human cardiac fibroblasts and the mechanisms of regulation of its target genes. We examined *miR-19a-3p/19b-3p* expression in human cardiac tissues and human cardiac fibroblasts by Q-PCR assay, as previously described. We integrated Targetscan 7.0 predictions and the resulting candidate functions and found that the TGF-β R II gene had the highest recurrence rate as a potential target gene of *miR-19a-3p/19b-3p*. MiRNAs are likely to bind partially to homologous sequence of a target gene in the 3′UTR^53^. We found that over-expression of *miR-19a-3p/19b-3p* could significantly reduce TGF-β R II gene expression. Furthermore, we detected the TGF-β/Smad2 signaling transduction undergoing the over-expression of *miR-19a-3p/19b-3p*. These results suggested that *miR-19a-3p/19b-3p* can directly and negatively regulate TGF-β R II gene expression and inhibit the TGF-β/Smad2 signaling transduction activated by TGF-β1.

## Additional Information

**How to cite this article**: Zou, M. *et al.* Autophagy inhibition of *hsa-miR-19a-3p/19b-3p* by targeting TGF-β R II during TGF-β1-induced fibrogenesis in human cardiac fibroblasts. *Sci. Rep.* **6**, 24747; doi: 10.1038/srep24747 (2016).

## Supplementary Material

Supplementary Information

## Figures and Tables

**Table 1:** Characteristics of heart failure patients (HF group) and healthy controls (C group).

Characteristics	HF group	C group	*p*
N(F/M)	20 (8/12)	20 (10/10)	>0.05
Age(years)	58.1 (10.1)	54.3 (12.4)	>0.05
LVEF (%)	37.1 (8.3)	64.8 (3.8)	<0.001
Log[BNP(ng/L)]	3.41 (0.32)	NA	NA

Values are expressed as mean (standard deviation).

F/M, numbers of females and males; LVEF, left ventricular ejection fraction as assessed by echocardiography; BNP, plasma brain natriuretic polypeptide (ng/L), NA, not available.
