# Supplementary material for: Autophagy inhibition of hsa-miR-19a-3p/19b-3p by targeting TGF-β R II during TGF-β1-induced fibrogenesis in human cardiac fibroblasts
Source: Sci Rep. 2016 Apr 21;6:24747. doi: 10.1038/srep24747 (PMC4838850; doi:10.1038/srep24747)
Supplement: Supplementary Information [file srep24747-s1.doc]

**Autophagy inhibition of *hsa-******miR-19a-3p/19b-3p* by targeting** **TGF-****β R Ⅱ during** **TGF****-β1-induced fibrogenesis in****human cardiac fibroblasts**

**Short title:** *MiR-19a-3p/19b-3p* inhibits autophagy-mediated fibrogenesis

Meijuan Zou^1^, Fang Wang^2^, Rui Gao^1^, Jingjing Wu^3^, Yingwei Ou^1^, Xuguan Chen^1^, Tongshan Wang^4^, Xin Zhou^4^, Wei Zhu^4^*, Ping Li^5^, Lian-Wen Qi^5^, Ting Jiang^6^, Weiwei Wang^6^, Chunyu Li^6^, Jun Chen^6^, Qifang He^6^, Yan Chen^6^*

^1^Department of Pharmacology, School of Basic Medical Sciences, Nanjing Medical University, 140 Hanzhong Road, Nanjing 210029, PR China;

^2^Department of Cardiology, First Affiliated Hospital of Nanjing Medical University, 300 Guangzhou Road, Nanjing 210029, PR China;

^3^Department Of Nephrology, First Affiliated Hospital of Nanjing Medical University, 300 Guangzhou Road, Nanjing 210029, P.R. China

^4^Department of Oncology, First Affiliated Hospital of Nanjing Medical University, 300 Guangzhou Road, Nanjing 210029, PR China;

^5^State Key Laboratory of Natural Medicines, China Pharmaceutical University, Nanjing 210009, China;

^6^Emergency Center, First Affiliated Hospital of Nanjing Medical University, 300 Guangzhou Road, Nanjing 210029, PR China;

*Corresponding author: Prof. Wei Zhu and Prof. Yan Chen.

Email address: [molecularnjmu@163.com](mailto:molecularnjmu@163.com), [zhuwei@njmu.edu.cn](mailto:zhuwei@njmu.edu.cn) and [chenyandoc@163.com](mailto:chenyandoc@163.com). Telephone: +86-25-68136428, +86 139 1389 4911, +86-25-68136930; Fax: +86-25 68136099.


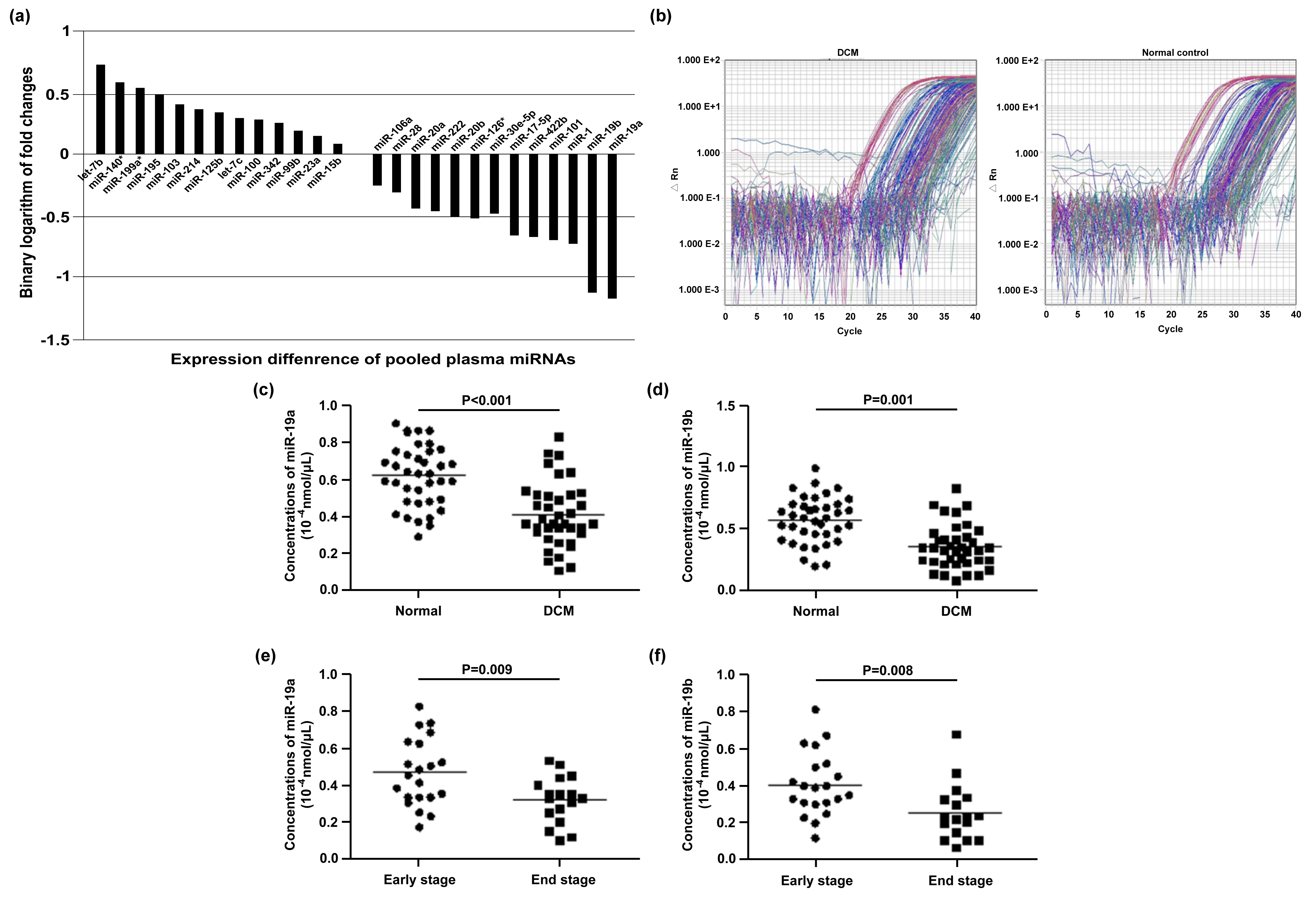


**Figure S1 *MiR 19a-3p/19b-3p* expresses with low levels in DCM patients and the expression level of patients in final edge is significantly lower than that in the early stage*.*** **(a)** The twenty plasma samples of 20 cases (10 cases of male and 10 cases of female, respectively) patients with dilated cardiomyopathy were randomly selected as DCM plasma pool samples; and another twenty plasma samples of 20 cases (10 cases of male and 10 cases of female, respectively) normal people were selected as normal control plasma pool samples. The differential expressions of 26 kinds of microRNAs from the literature were verified by qRT-PCR assay and ath-156a was added as the external reference for corresponding control. The expressions of miRNA 19a/b were significantly down-regulated in DCM plasma pool samples. **(b)** The amplification curves of DCM (left) and Normal control (right). **(c) and (d)** The expression of miRNA 19a/b in the plasma samples of 38 patients with DCM was quantitatively tested by qRT-PCR. miRNA 19a/b decreased in DCM plasma samples. P < 0.001 (miRNA 19a); P = 0.001 (miRNA 19b). **(e) and (f)** The expression of miRNA 19a/b in the plasma samples of patients with different stage of DCM was quantitatively tested by qRT-PCR. miRNA 19a/b decreased more obviously in end stage of DCM plasma samples than in early stage. P = 0.009 (miRNA 19a); P = 0.008 (miRNA 19b).
